# Characterization of m6A Regulator-Mediated Methylation Modification Patterns and Tumor Microenvironment Infiltration in Ovarian Cancer

**DOI:** 10.3389/fcell.2021.794801

**Published:** 2022-01-11

**Authors:** Yihong Luo, Xiang Sun, Jian Xiong

**Affiliations:** Department of Gynaecology and Obstetrics, Guangzhou Women and Children’s Medical Center, Guangzhou Medical University, Guangzhou, China

**Keywords:** ovairan cancer, N6-methyladenosine, microenvironment, RNA methylation, immunotherapy

## Abstract

**Introduction:** Studies have demonstrated the epigenetic regulation of immune responses in various cancers. However, little is known about the RNA N6-methyladenosine (m6A) modification patterns of the microenvironment (TME) cell infiltration in ovarian cancer (OC).

**Methods:** We evaluated the correlation between m6A modification patterns and TME cell infiltration based on 459 OC samples from the Cancer Genome Atlas and Gene-Expression Omnibus database. We constructed an m6Ascore system to quantify m6A modification patterns using principal component analysis.

**Results:** Based on unsupervised clustering, three m6A modification patterns were identified. Gene set variation analysis showed that the antigen presentation signal pathway, the NOTCH signaling pathway, and the metabolism-related pathway differed significantly across m6A modificaiton patterns. The m6Ascore is closely correlated with TME cell infiltration. OC patients with lower m6Ascores had worse outcomes. There was better risk stratification with combined m6Ascore and tumor mutation burden. The responses to immune checkpoint inhibitor treatment significantly differed between high and low m6Ascore groups.

**Conclusion:** M6A modification plays an essential role in TME cell infiltration in OC. Evaluating the m6A modification patterns in OC patients could enhance our understanding of TME infiltration characterization and guide immunotherapy strategies.

## Introduction

Ovarian cancer (OC) is the third most common cancer in the female reproductive system and the leading cause of cancer-related death among gynecological cancers (Siegel et al., 2018). Patients with OC are often diagnosed at an advanced stage because of a lack of early diagnosis methods. There remains a lack of satisfactory treatment for patients with advanced OC. Thus, OC patients suffer poor outcomes and high relapse rates. The 5-year survival for most OC patients is less than 40% despite the advances in therapies such as adjuvant chemotherapy and cytoreductive surgery (Armstrong et al., 2020). Therefore, understanding the molecular mechanisms underlying the pathogenesis and development of OC may advance the diagnosis and treatment of OC.

Several lines of evidence suggested that epigenetics plays an essential role in ovarian carcinogenesis. N6-methyladenosine (m6A) RNA methylation, one of the most dominant drivers of eukaryotic mRNA modification, is a common form of epigenetic regulation (Liu and Pan, 2016). Many proteins defined as m6A “writers” (METTL3, METTL14, WTAP, et al.), “readers” (YTHDF1, YTHDF2, YTHDC2, HNRNPA2B1, et al.) and “erasers” (ALKBH5 and FTO) participate in the modification of m6A methylation (Roundtree et al., 2017). An in-depth understanding of these m6A regulators would contribute to uncover the mechanism of m6A RNA modification in post-transcriptional regulation. Evidence revealed that m6A regulators are implicated in disorders of diverse biological processes, including cell proliferation and death, embryonic stem cell self-renewal, fate determination, cancer progression, and immunomodulatory abnormalities (Yue et al., 2015; Deng et al., 2018). For instance, the m6A writer METTL3 can promote the growth and invasion of OC via stimulating AXL translation (Hua et al., 2018). The m6A reader YTHDF1 promotes the progression of OC via augmenting the translation of EIF3C in a m6A dependent manner (Liu et al., 2020). The m6A eraser FTO can inhibit the self-renewal of OC cancer stem cell via blocking cAMP signaling (Huang et al., 2020).

Studies demonstrated that the tumor microenvironment (TME) plays an essential role in tumor progression. TME is constructed by stromal cells, containing fibroblasts, mesenchymal stem cells, and immune cells (Quail and Joyce, 2013). Innate immune cells (neutrophils, dendritic cells, macrophages, innate lymphoid cells, natural killer cells, and myeloid-derived suppressor cells) and adaptive immune cells (B cells and T cells) in the TME contribute to tumor progression (Hinshaw and Shevde, 2019). TME components directly and indirectly affect multiple biological behaviors of cancer cells such as inhibiting apoptosis, inducing proliferation, avoiding hypoxia, inducing immune tolerance, et al. (Binnewies et al., 2018). The evidence suggests that m6A modification could build a TME favorable for the growth of cancer cells (Zhu et al., 2021). For instance, Yi Jian et al. found that ALKBH5 could promote the progression of OC in a simulated TME via NF-κB signaling (Jiang et al., 2020). Therefore, a comprehensive understanding of the correlation between TME and m6A regulators might help elucidate the mechanisms of TME immune regulation. In the present study, we integrated the genomic data of OC samples from the Cancer Genome Atlas (TCGA) and Gene-Expression Omnibus (GEO) to evaluate the m6A modification pattern and its correlation with TME, which would enhance our understanding of how m6A modification participates in shaping TME in OC.

## Methods

### Data Source

The mRNA expression profiles and clinical data containing 379 OC samples were downloaded from TCGA (http://cancergenome.nih.gov/). GSE14764 (Denkert et al., 2009) containing 80 OC samples was downloaded from GEO (http://www.ncbi.nlm.nih.gov/geo/). Samples without survival data were removed from further analysis. The “ComBat” algorithm of the sva package was used to correct the batch effects from non-biological technical biases. The somatic mutation data were downloaded from TCGA. Data in this study were analyzed using R software (version 3.6.1) and R Bioconductor packages.

### Unsupervised Clustering for 24 m6A Regulators

A total of 24 m6A regulators were extracted to identify distinct m6A modification patterns. Based on the expression of these 24 m6A regulators, unsupervised clustering analysis was used to identify different m6A modification patterns, and classify OC patients for further analysis. A consensus clustering algorithm was used to determine the number of clusters and their stability (Wong, 1979). The ConsensusClusterPlus package was used to perform this algorithm, and we conducted 1000 times repetitions to guarantee the stability of clusters (Hayes, 2010).

### Gene Set Variation Analysis

GSVA enrichment analysis was performed to investigate the biological processes between different m6A clusters. GSVA is commonly used to estimate the variation in biological processes in an expression dataset (Hänzelmann et al., 2013). We downloaded the gene set of “c2.cp.kegg.v6.2.symbols” from MSigDB for GSVA analysis. The clusterProfiler R package was used to conduct functional annotation for m6A-related genes. The cut-off value of FDR was 0.05. Adjusted *p* < 0.05 was represented statistical significance.

### Tumor Microenvironment Cell Infiltration

We used single-sample gene set enrichment analysis (ssGSEA) to quantify the relative abundance of each immune cell infiltration in the OC TME. The gene set for marking each immune cell type was as described previously (Charoentong et al., 2017). The relative abundance of each immune infiltrating cell in each sample was represented with enrichment scores.

### Construction of the m6A Gene Signature

To identify m6A-related genes, the OC samples were classified into three m6A modification patterns according to the expression of m6A regulators. Differentially expressed genes (DEGs) between m6A modification patterns were identified using the empirical Bayesian approach of the limma package. DEGs with adjusted *p* < 0.001 were considered significant.

We constructed an m6A gene signature (the m6Ascore) to quantify m6A modification patterns. The overlap of DEGs from various m6A clusters was extracted. We classified the OC patients into several groups using an unsupervised clustering method to analyze the overlap DEGs. The number of gene clusters and their stability were defined using the consensus clustering algorithm. Then, we extracted the genes with significant outcomes using a univariate Cox regression model. Principal component analysis (PCA) was performed to construct an m6A gene signature. The m6Ascore is defined as: m6Ascore = $\sum \left(\mathrm{PC}1_{i}+\;\mathrm{PC}2_{i}\right)$ , where i represents each of the m6A-related genes (Zhang et al., 2020).

### The Association Between m6A Gene Signature and Immunotherapy

The immunophenoscores (IPSs) of OC patients were downloaded from the cancer-immune group atlas (TCIA, https://tcia.at/home). The IPS was obtained according to four categories of immunogenicity-related genes (effector cells, MHC molecules, immunosuppressor cells, and immune modulators). The value of IPS ranges from 0 to 10, calculated based on z-scores, representing the expression of genes in cell types. The values of IPS are positively correlated with immunogenicity. A correlation analysis was also conducted to reveal the association between m6Ascore and TME.

### Cell Culture and Transfection

Human ovarian cancer cell lines, A2780 and OVCAR3, were both obtained from the Chinese Academy of Sciences Cell Bank (China). A2780 and OVCAR3 were cultured in RPMI 1640 (Hyclone) and DMEM (High-Glucose) medium (Hyclone) supplemented with 10% serum. Both cells were cultured at 37°C in an atmosphere of 5% CO2.

### Transwell Assay

Migration and invasion of ovarian cancer cells were measured by transwell chamber with 8-μm pores (Corning Costar, Corning, NY, United States) using 24-well plates. Briefly, A2780 and OVCAR3 cells (5 × 104) in 300 µl serum-free culture media were added into the upper chamber with 10% FBS in the lower chamber inserted in 24-well plates and cultured for 8 h. The migrated cells on the lower side of the membranes were fixed with methanol and then stained with crystal violet and counted.

### Wound-Healing Migration Assay

Wound-healing assay was used to measure the migration ability of cells. Cells were seeded into six-well plates and a straight line was drawn with a 1,000-µl sterile pipette when the density was 90–100%. Incubate for 48 h after the addition of serum-free medium. A microscope was used to make photography of scratch at 0 and 48 h.

### Statistical Analysis

Spearman and distance correlation analyses were used to compute the correlation coefficients between the expression of m6A regulators and TME infiltrating immune cells. The *t*-test and Kruskal-Wallis tests were used for comparisons between two groups. Comparisons of three or more groups were conducted using one-way ANOVA or Kruskal-Wallis tests. The Kaplan-Meier method was used to generate the survival curves for predictive analysis, and the significance of differences was identified using the log-rank test. The survminer R package was used to determine the cut-off point for each subgroup. A multivariable Cox regression analysis was conducted to identify the independent prognostic factors. The waterfall function of the maftools package was used to generate the mutation landscape. The copy number variation landscape was plotted using the RCircos R package. All data were processed in R 3.6.1 software with a *p*-value < 0.05, indicating statistical significance.

## Results

### Genetic Variation and Survival Analysis of m6A Regulators in Ovarian Cancer

**FIGURE 1:**
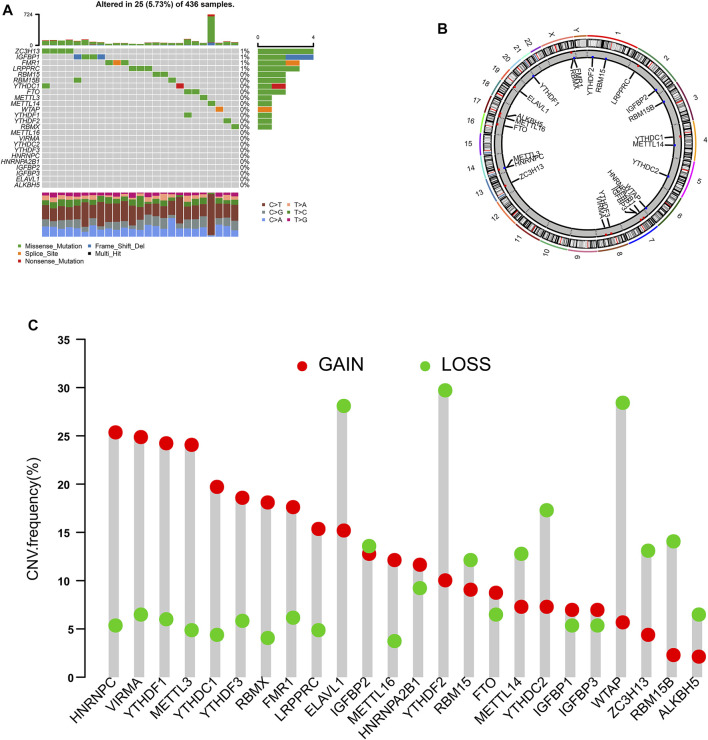
Landscape of genetic variation of m6A regulators in ovarian cancer. **(A)** The mutation frequency of 24 m6A regulators in patients with ovarian cancer from TCGA cohort. **(B)** The location of CNV alteration of m6A regulators on 23 chromosomes using TCGA cohort. **(C)** The CNV variation frequency of m6A regulators according to TCGA cohort.

**FIGURE 2:**
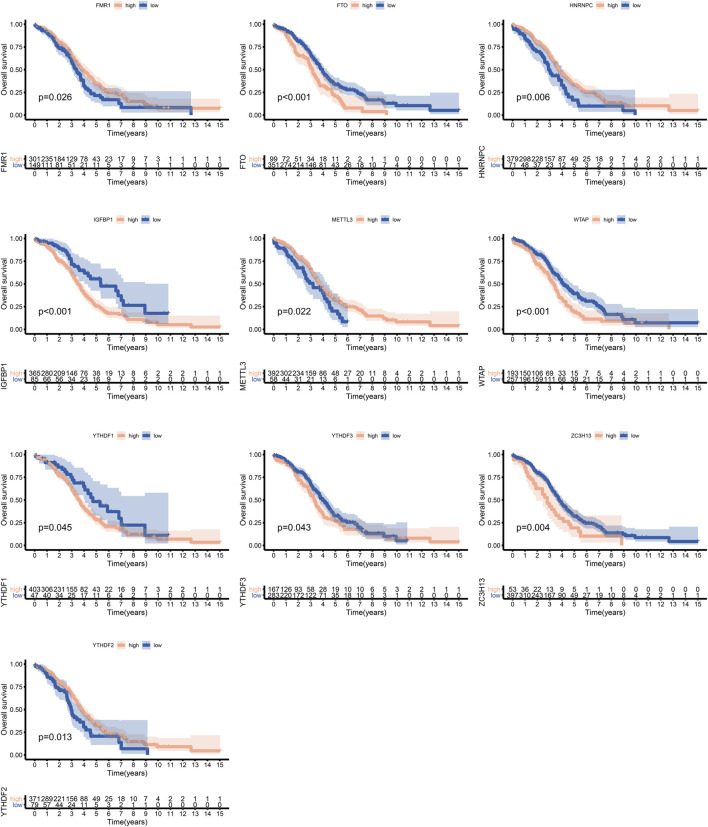
Survival analysis of m6A regulators in ovarian cancer according to TCGA and GSE14764 cohorts.

We identified 24 m6A regulators in this study. The incidences of somatic mutations and copy number variations (CNVs) of the 24 m6A regulators are summarized in Figure 1. Twenty-five of 436 samples experienced mutations of m6A regulators. ZC3H13 and IGFBP1 exhibited the highest mutation frequency, while METTL16, VIRMA, YTHDC2, YTHDF3, HNRNPC, HNRNPA2B1, IGFBP2, IGFBP3, ELAVL1, and ALKBH5 showed no mutations in OC samples (Figure 1A). Figure 1B shows the location of CNV alteration of the 24 m6A regulators on chromosomes. HNRNPC, VRMA, YTHDF1, METTL3, YTHDC1, YTHDF3, RBMX, FMR1, and LRPPPC were focused on the amplification in copy number, while ELAVL1, YTHDF2, and WTAP had a widespread frequency of CNV deletion. Among these m6A regulators, the expressions of FMR1, FTO, HNRNPC, IGFBP1, METTL3, WTAP, YTHDF1, YTHDF3, ZC3H13, and YTHDF2 were significantly correlated with the outcome of OC, according to TCGA and GSE14764 cohorts (Figure 2).

### Tumor Microenvironment Cell Infiltration in Different m6A Modification Patterns

**FIGURE 3:**
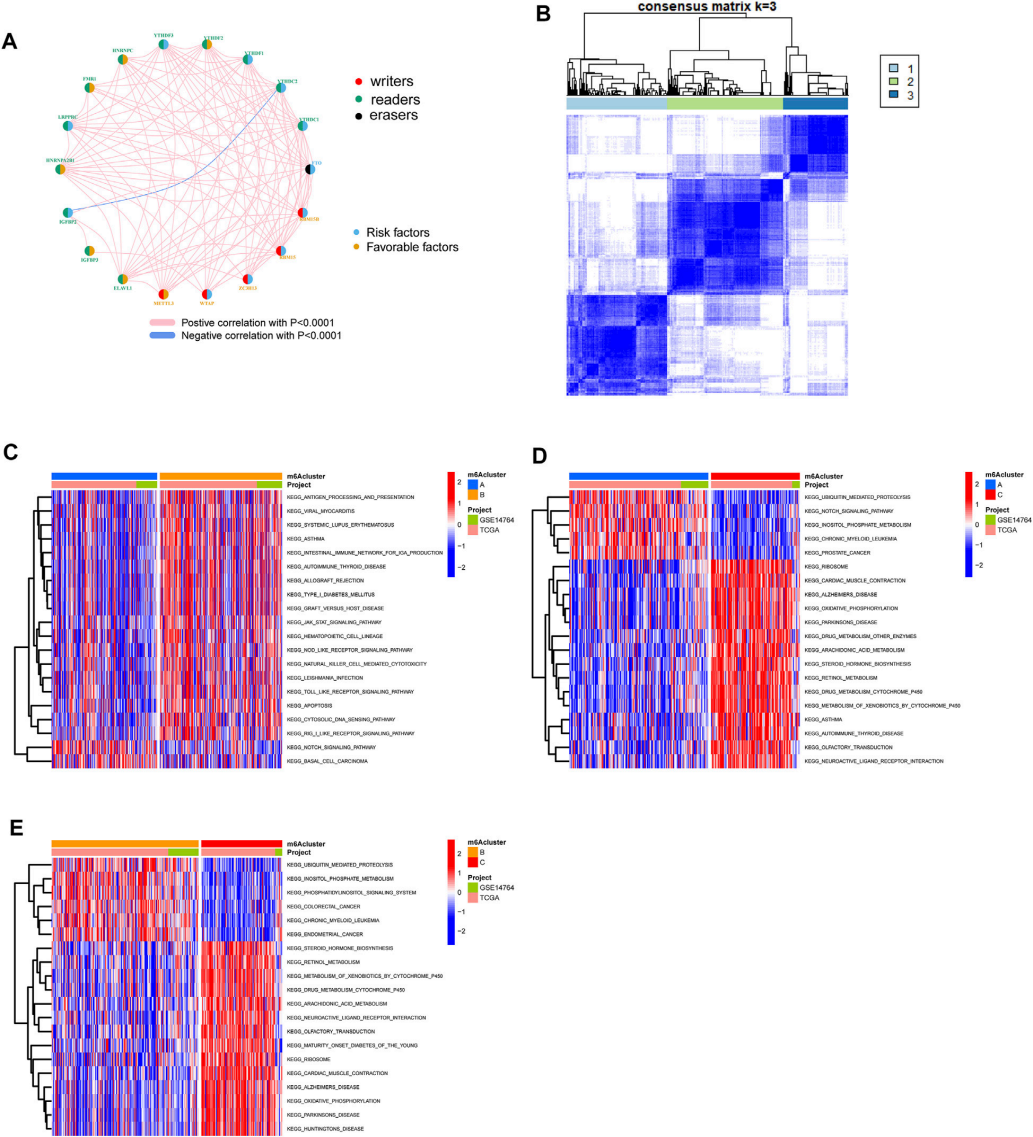
Patterns of m6A methylation modification and biological characteristics of each pattern. **(A)** The interaction between m6A regulators in ovarian cancer. **(B)** Consensus matrices of m6A regulators according to TCGA and GSE14764 cohort for k = 3. **(C–E)**. GSVA enrichment analysis showing the activation states of biological pathways in distinct m6A modification patterns.

**FIGURE 4:**
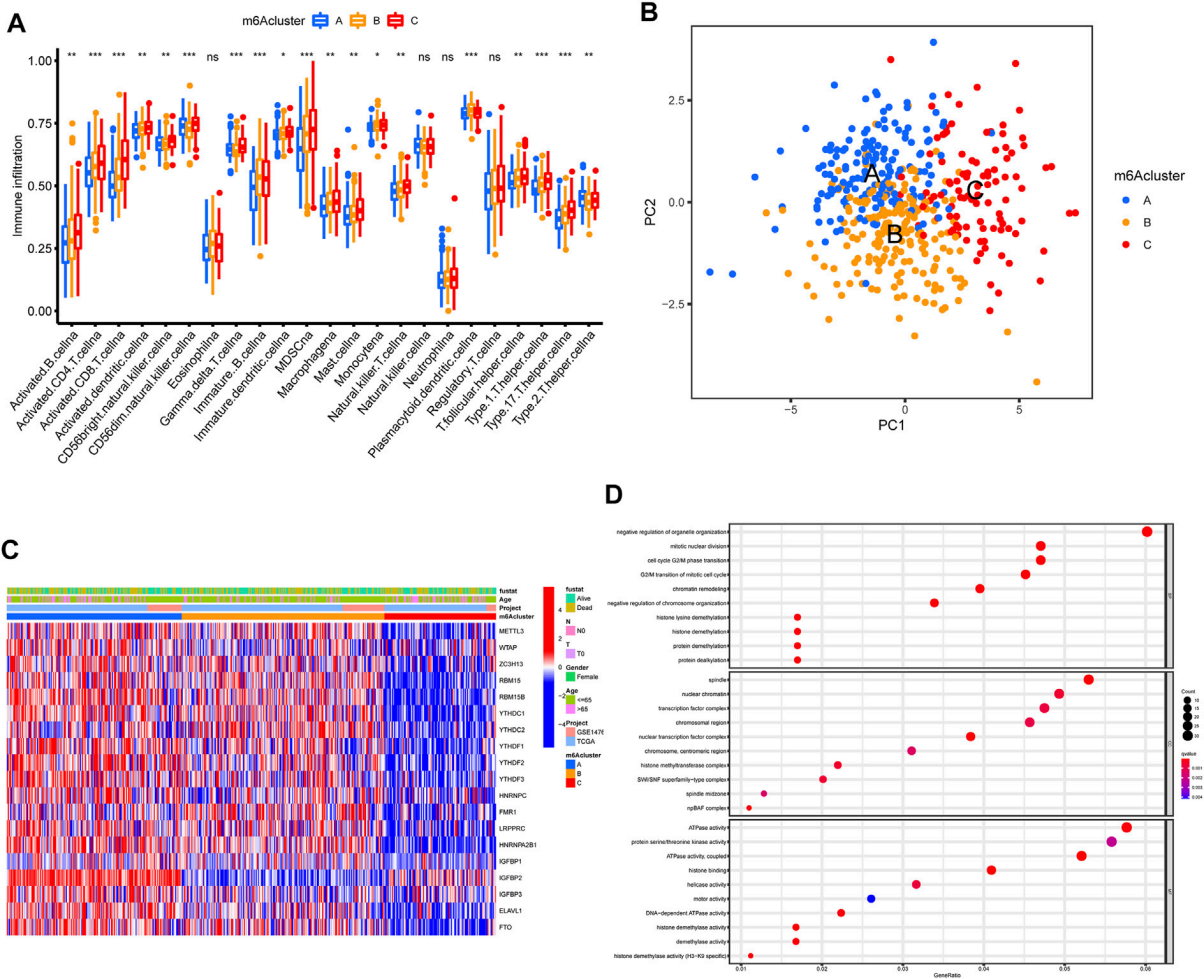
TME cell infiltration characteristics and transcriptome traits in distinct m6A modification patterns. **(A)** The abundance of each TME infiltrating cell in three m6A modification patterns. **(B)** Principal component analysis for the transcriptome profiles of three m6A modification patterns, showing a remarkable difference on transcriptome between different modification patterns. **(C)** Unsupervised clustering of m6A regulators according to TCGA and GSE14764 cohort. **(D)** Functional annotation for m6A-related genes using GO enrichment analysis.

The OC patients were classified with different m6A modification patterns based on the expression of m6A regulators using the ConsensusClusterPlus R package. Unsupervised clustering was used to identify three distinct m6A modification patterns, called m6Acluster A–C (Figure 3B, Figure 4C). GSVA enrichment analysis was performed to analyze the biological process among different m6Aclusters (Figures 3C–3E). Subsequent analysis of TME cell infiltration showed that m6Acluster-C was significantly rich in activated CD4/8 T cell, MDSC, macrophage, regulatory T cells, et al. (Figure 4A). The M6A score and the results of six different immune infiltration assessments are presented in Supplementary Figure S1.

### Construction of an m6A Gene Signature

**FIGURE 5:**
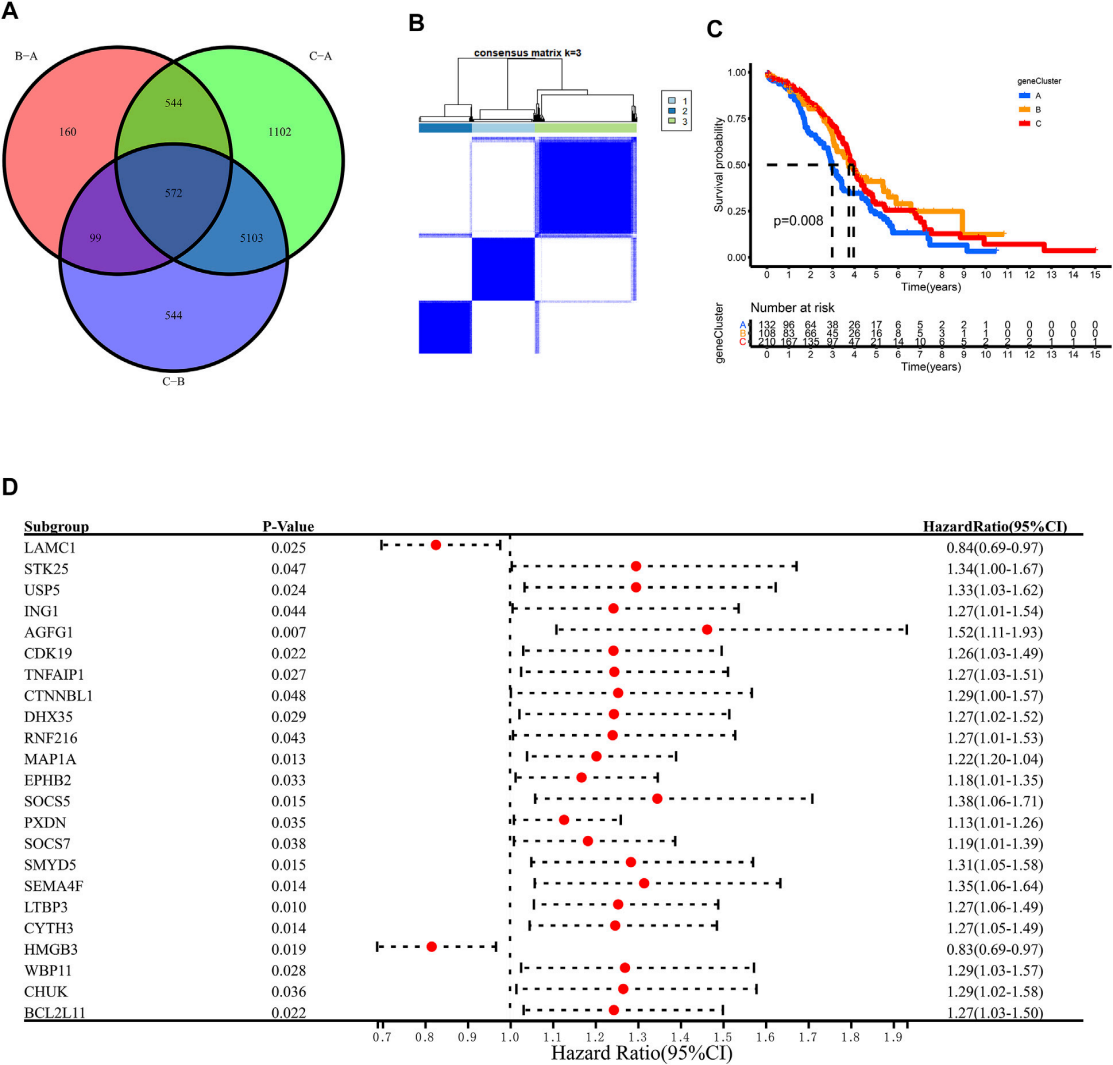
Unsupervised clustering of 572 m6A phenotye-related genes according to TCGA and GSE14764 cohort. **(A)** 572 m6A phenotype-related genes shown in venn diagram. **(B)** Consensus matrices of 572 m6A phenotye-related genes according to TCGA and GSE14764 cohort for k = 3. **(C)** Survial analysis for the three m6A modification patterns in TCGA and GSE14764 cohort. **(D)** The prognostic analysis for m6A phenotye-related genes in TCGA and GSE14764 cohort using a multivariate Cox regression model.

**FIGURE 6:**
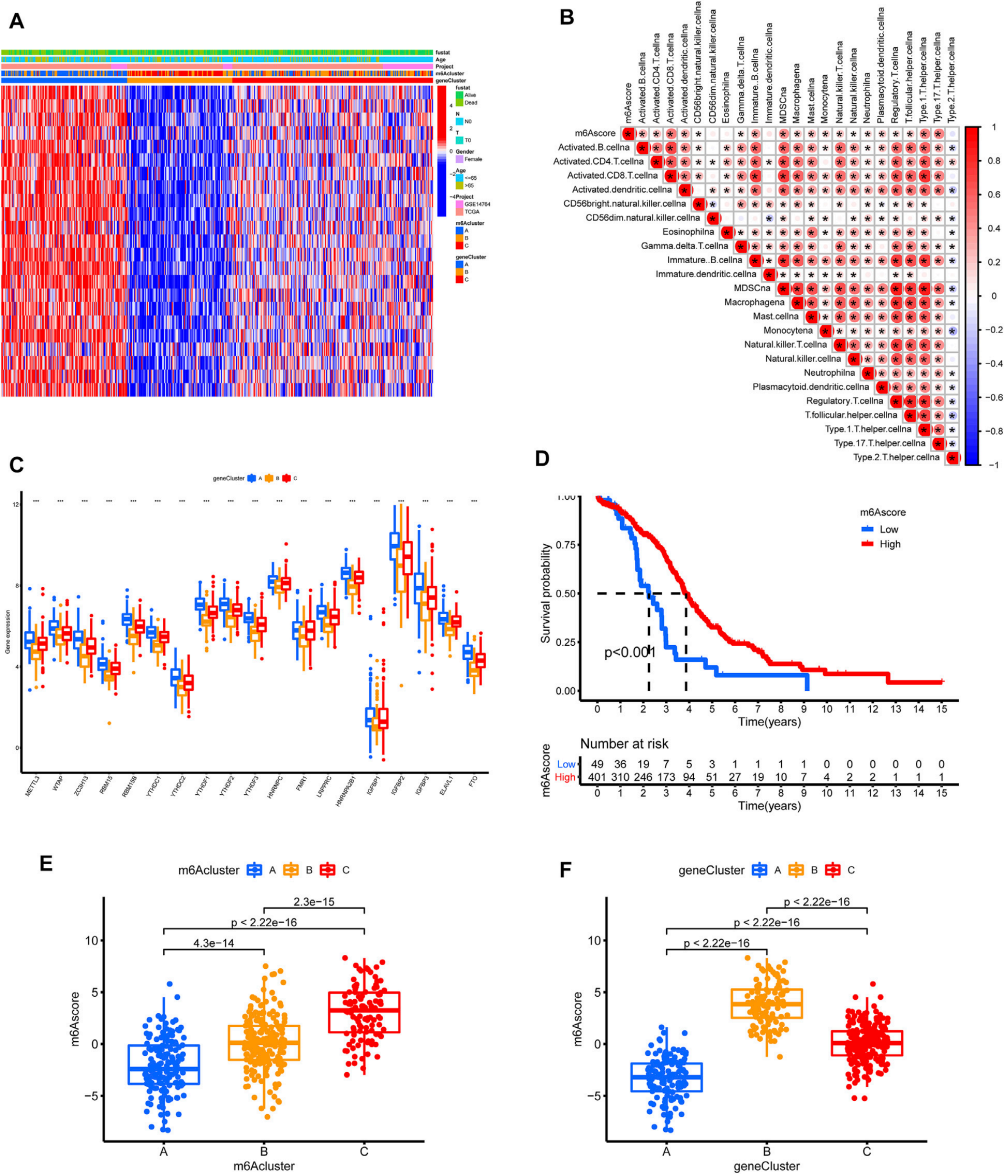
Construction of m6A signature according to TCGA and GSE14764 cohort. **(A)** Unsupervised clustering of the m6A phenotype-related genes to classify patients into different genomic subtypes. **(B)** Correlations between m6Ascore and the TME infiltrating cell using Spearman analysis. **(C)** The expression of m6A regulators in three gene clusters. **(D)** Survival analysis for low and high m6Ascore patient groups using Kaplan-Meier curves. **(E,F)** Comparison of m6Ascores in different m6Aclusters and geneclusters.

To further explore the m6A modification pattern, 572 m6A phenotype-related DEGs were extracted from three distinct m6Aclusters (Figure 5A). A multivariable Cox regression was conducted to identify genes with independently prognostic values (Figure 5D). GO enrichment analysis was performed to identify the biological processes related to 572 DEGs (Figure 4D). Then, we classified the OC patients into different genomic subtypes based on the 572 m6A phenotype-related DEGs using unsupervised clustering analysis. The OC patients were classified into three m6A modification genomic phenotypes, called genecluster A–C (Figure 5B, Figure 6A). This indicated that three distinct m6A modification patterns did exist in OC. The three geneclusters were closely associated with m6Aclusters (Figure 6A). The expression levels of m6A regulators differed significantly in the three geneclusters, following our expectation based on the m6A modification patterns.

Considering the complexity and heterogeneity of m6A modification, we constructed a scoring system to quantify m6A modifications based on the 572 m6A phenotype-related genes, termed as the m6Ascore. To better illustrate m6Ascore characteristics, we tested the correlations between the m6Ascore and TME cell infiltration (Figure 6B). The expression difference of M6A regulators in the three subgroups were shown in Figure 6C. A survival analysis revealed that patients with lower m6Ascores suffered from worse outcomes (Figure 6D). The m6Ascores significantly differed across m6Aclusters and geneClusters (Figures 6E,F).

### Characteristics of m6A Modification and Tumor Somatic Mutation

**FIGURE 7:**
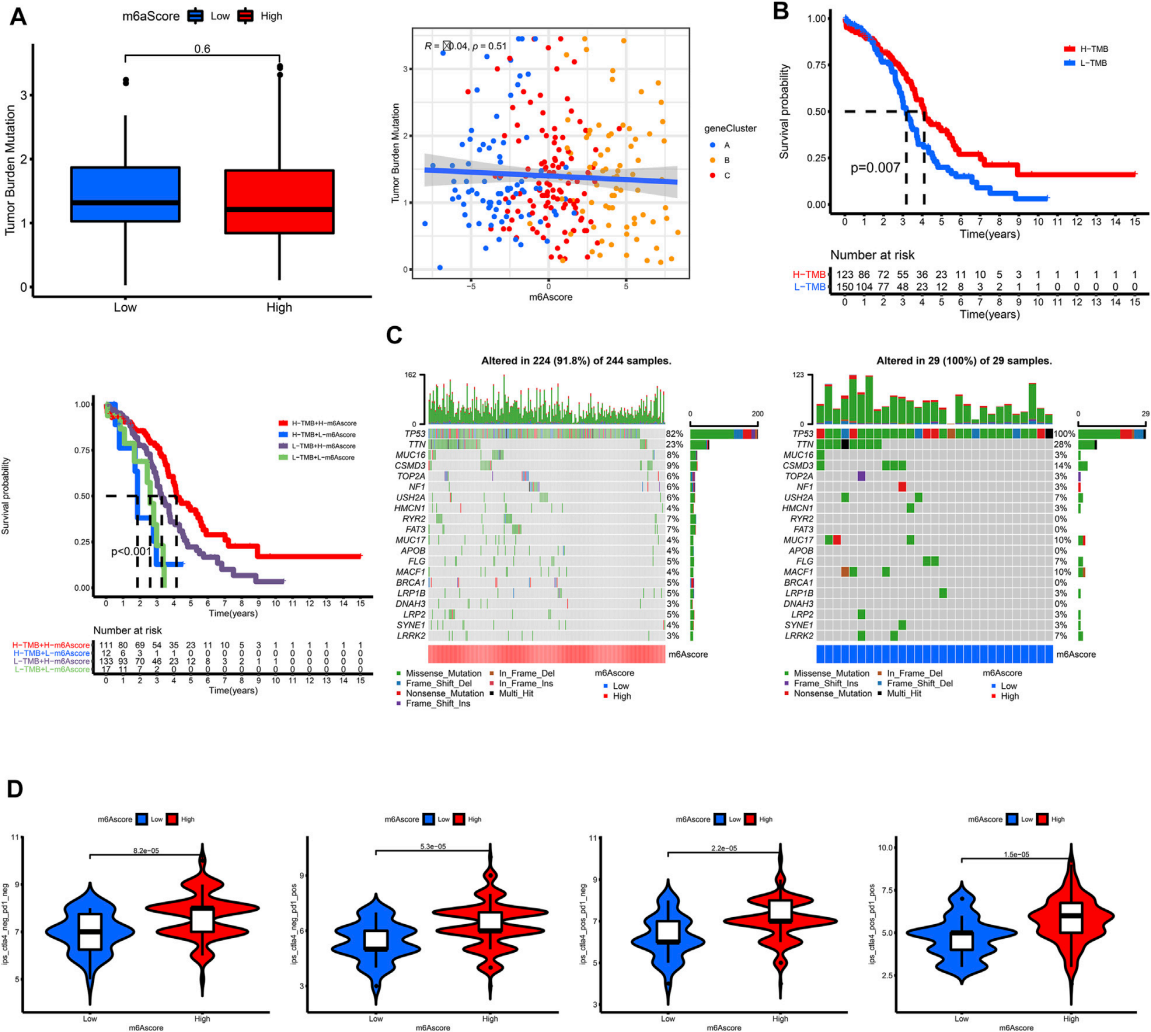
Characteristics of m6A modification and tumor somatic mutation in TCGA. **(A)** The correlation of TMB and m6Ascore. **(B)** Survival analysis for subgroup patients stratified by both TMB and m6Ascore using Kaplan-Meier curves. **(C)** The waterfall plot of tumor somatic mutation established by those with high m6Ascore and low m6Ascore. **(D)** The association between m6Ascore and immunotherapy.

According to OC samples in TCGA, we found that OC patients with a lower tumor mutation burden had worse outcomes (Figure 7B). However, the m6Ascore was not correlated with tumor mutation burden (Figure 7A). Interestingly, it provided a better risk stratification combining m6Ascore with tumor mutation burden (Figure 7B). We further analyzed the distribution of somatic mutation between high and low m6Ascore groups, showing that the tumor mutation burden was of no significant difference between high and low m6Ascore groups (Figure 7C).

### The Association Between m6A Gene Signature and Immunotherapy

A correlation analysis was also conducted to reveal the associations between m6Ascore and immunotherapy. The OC patients with higher m6Ascore had higher IPS in all of the CTLA4_neg_PD1_neg, CTLA4_neg_PD1_pos, CTLA4_pos_PD1_neg, and CTLA4_pos_PD1_pos groups, indicating that these patients would have a better response to immunotherapy.

### Knockout of YTHDF2 Significantly Inhibits Ovarian Cancer Cell Migration and Invasion

**FIGURE 8:**
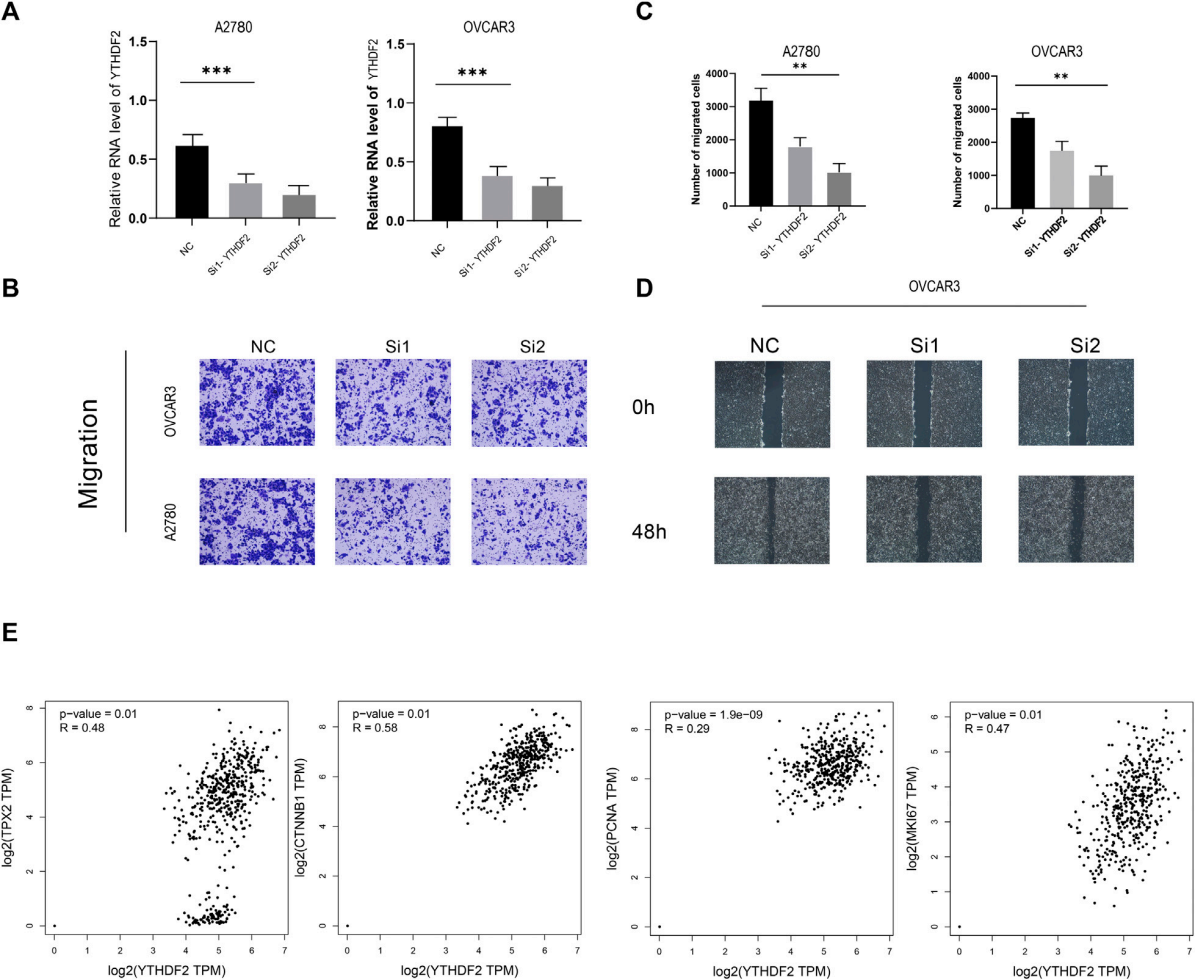
A2780 and OVCAR3 Cell experiments. **(A)** RNA level of YTHDF2 in A2780 and OVCAR3 Cell experiments following YTHDF2 knockdown. **(B,C)** Knockdown of YTHDF2 decreased cell migration as detected by Transwell assays. **(D)** Knockdown of YTHDF2 decreased cell migration as detected by Wound-healing migration assays. **(E)**. YTHDF2 shows positive correlation with TPX2, MKI67, PCNA and CTNNB1.

To further explore the relationship between YTHDF2 and cell proliferation and invasion phenotype in ovarian cancer cell lines, we conducted a series experiments *in vitro*. Transwell experiment proved that the migration ability of YTHDF2-knockdown group was significantly lower than that of NC group (Figures 8A–C). The wound healing test is used to study the effect of YTHDF2 on the migration of ovarian cancer cells. The results showed that the reduction of YTHDF2 led to the weakening of the migration ability of ovarian cancer cells (Figure 8D). Later, we gave the TCGA cohort and found that the expression level of YTHDF2 has a close positive correlation with common cell proliferation-related proteins, such as MKI67, PCNA, CTNNB1, TPX2 (Figure 8E).

## Discussion

It appears that m6A modification participates in the occurrence and development of tumors by interacting with a variety of m6A regulatory factors. A variety of m6A regulatory factors have been reported in the literature. Therefore, we identified groups of m6A regulatory modes in OC using transcriptional sequencing data and determined the biological functions of groups of various m6A regulatory modes using functional analysis. Adjust the difference in pathways. We then identified DEGs of m6A regulators using pairwise difference analysis. These genes were later used to construct the m6Ascore.

We identified three m6A regulatory expression patterns based on unsupervised clustering. The GSVA score of the antigen presentation signal pathway in the C1 group is relatively low, suggesting that m6A in OC may affect the process of antigen presentation in the immune system. Han et al. found that mRNA m6A methylation mediates durable neoantigen-specific immunity after being regulated by the m6A binding protein YTHDF15 (Han et al., 2019). Compared with wild-type mice, ythdf1-deficient mice showed a higher antigen-specific CD8^+^ T cell anti-tumor response. The deletion of YTHDF1 in classical dendritic cells enhances the cross-presentation of tumor antigens and the cross-priming of CD8^+^ T cells *in vivo*. The NOTCH signaling pathway GSVA score was higher in the C1 group, suggesting that m6A significantly affects the NOTCH signaling pathway in OC. Zhang et al. found that continuous activation of Notch signal in METTL3-deficient embryonic arterial endothelial cells blocked the endothelial-to-haematopoietic transition (EHT) and inhibited the production of the earliest hematopoietic stem and progenitor cells (Zhang et al., 2017).

Colon cancer and endometrial cancer in the C2 group have higher GSVA scores, suggesting that m6A in OC may affect the regulation process of colon cancer and endometrial cancer pathways. IGF2BP3, METTL3, ALKBH5, et al. have been shown to be related to the proliferation and invasion of colon cancer tumor cells (Xu et al., 2020; Yang et al., 2020; Guo et al., 2020)–(Guo et al., 2020; Xu et al., 2020; Yang et al., 2020). IGF2BP and FTO are associated with tumorigenesis of endometrial epithelial carcinoma (Zhang et al., 2021a; Zhang et al., 2021b). In addition, the GSVA score of the oxidative phosphorylation signaling pathway in the C2 group is lower, suggesting that m6A may significantly affect the oxidative phosphorylation signaling pathway in OC. Pastò et al. Found that oxidative phosphorylation in stem cells of patients with epithelial OC (Pastò et al., 2014). Later, oxidative phosphorylation was also confirmed as a therapeutic target for OC (Burnett, 2019).

The GSAV scores of metabolism-related pathways in the C3 group are relatively high, suggesting that m6A may affect the metabolic process in the body in OC. Dongjun Dai et al. believe that m6A modification affects almost every step of RNA metabolism, including mRNA processing, mRNA output from the nucleus to the cytoplasm, mRNA translation, mRNA decay, and the biogenesis of long non-coding RNA (lncRNA) and microRNA (miRNA) (Zhao et al., 2017; Dai et al., 2018).

We found that m6Ascore is related to immune microinfiltration, and there are differences in different immune checkpoint inhibitor treatment response groups, suggesting that M6A plays an essential role in the tumor microenvironment. The role of m6A in dendritic cells (DCs) has been elucidated. DNA and RNA trigger innate immune responses by activating toll-like receptors (TLRs) on the surface of DCs. However, when DCs were exposed to m6A-modified RNA, compared with unmodified RNA, cytokines and activation markers were significantly produced, indicating that m6A modification hindered the activation of DCs. In addition, the innate immune system can detect RNA lacking m6A as a means of selectively responding to bacteria or necrotic tissue (Karikó et al., 2005). In addition, the methylation of m6A on mRNA controls the homeostasis of T cells. Studies showed that reduced m6A levels (caused by Mettl3-KO) lead to reduced Socs mRNA degradation, leading to inactivation of the IL-7/STAT5/Socs pathway. IL-7 stimulation activates the JAK/STAT pathway through m6A regulation, and downregulates the expression of SOCS family genes, and initiates the reprogramming of naive T cells to achieve differentiation and proliferation (Li et al., 2017).

There are some limitations to this article. Although the two cohorts were combined for data analysis, our findings of the article have not been verified by basic experiments and clinical trials. These limitations will be addressed in our future work.

## Conclusion

There are three m6A mediation modes with significantly different representative pathways. We identified these representative genes according to the grouping results of these genes, an m6Ascore was constructed. The m6Ascore was closely related to the immune microenvironment and immunotherapy response. We provided a comprehensive analysis of the m6A regulation mode and biological effects in OC. These findings may provide support for the study of the mechanism of m6A mediation mode in OC.

## Data Availability

The datasets presented in this study can be found in online repositories. The names of the repository/repositories and accession number(s) can be found in the article/Supplementary Material.
