# Supplementary figures and images for: Characterization of m6A Regulator-Mediated Methylation Modification Patterns and Tumor Microenvironment Infiltration in Ovarian Cancer
Source: Front Cell Dev Biol. 2022 Jan 11;9:794801. doi: 10.3389/fcell.2021.794801 (PMC8787330; doi:10.3389/fcell.2021.794801)

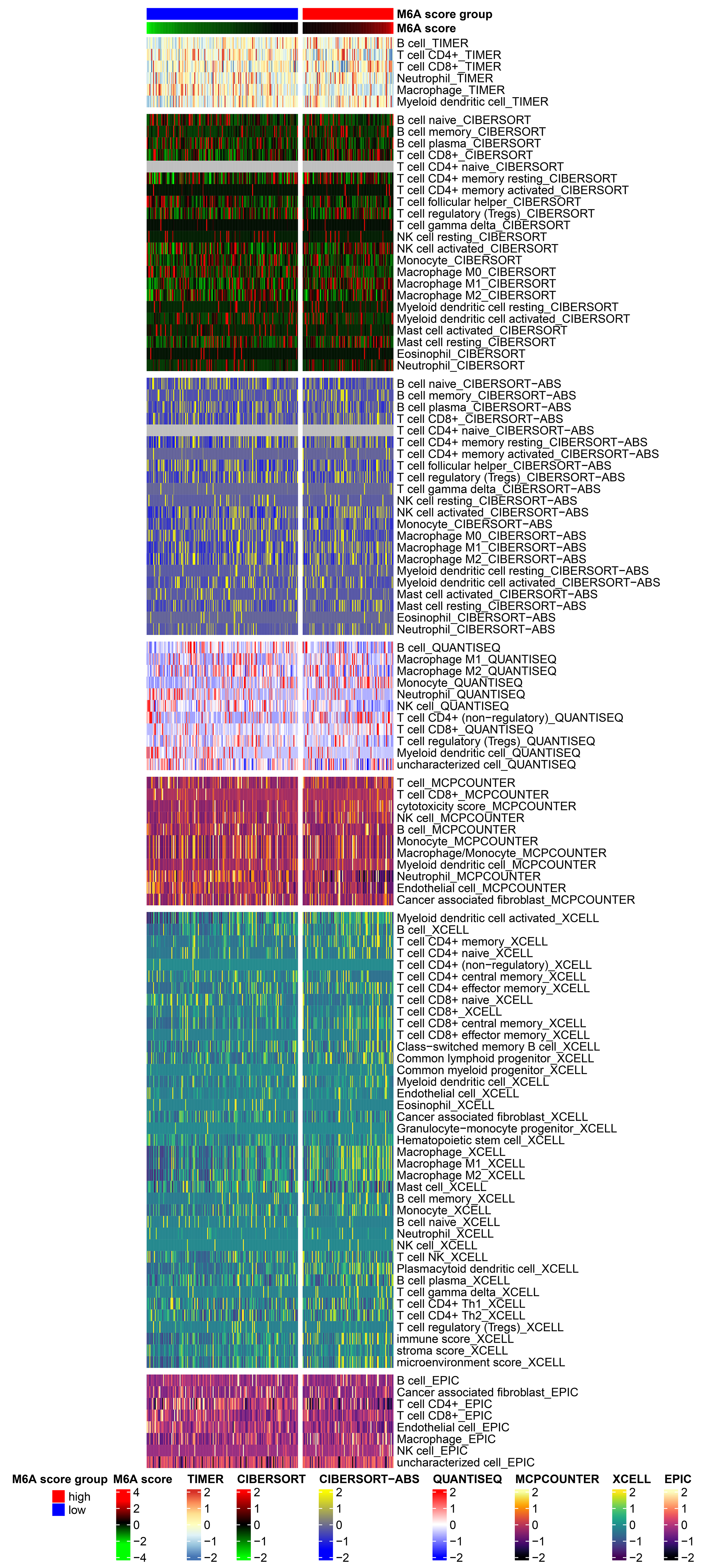

Supplement: Supplementary file 1 [file Image1.TIF]
